# Commentary on ‘Endovascular treatment versus standard medical treatment in patients with established large infarct: a cohort study’

**DOI:** 10.1097/JS9.0000000000001699

**Published:** 2024-05-22

**Authors:** Olabisi O. Ogunleye, Ayush Anand, Mahalaqua N. Khatib, Abass O. Ajayi, Quazi S. Zahiruddin, Sarvesh Rustagi, Prakasini Satapathy, Nathnael A. Woldehana

**Affiliations:** a Department of Surgery, Abubakar Tafawa Balewa University, Bauchi; b Ahmadu Bello University Teaching Hospital, Zaria, Nigeria; c MediSurg Research, Darbhanga; d Division of Evidence Synthesis; e South Asia Infant Feeding Research Network (SAIFRN), Division of Evidence Synthesis, Global Consortium of Public Health and Research, Datta Meghe Institute of Higher Education, Wardha; f School of Applied and Life Sciences, Uttaranchal University, Dehradun, Uttarakhand; g Center for Global Health Research, Saveetha Medical College and Hospital, Saveetha Institute of Medical and Technical Sciences, Saveetha University, Chennai, India; h B.P. Koirala Institute of Health Sciences, Dharan, Nepal; i Medical Laboratories Techniques Department, Al-Mustaqbal University, Hillah, Babil, Iraq; j Myungsung Medical College, Addis Ababa, Ethiopia; k Johns Hopkins Bloomberg School of Public Health, Baltimore, Maryland, USA

*Dear Editor,*

We would like to congratulate Guo *et al*.^1^ for publication of the manuscript titled ‘Endovascular treatment versus standard medical treatment in patients with established large infarct: a cohort study.’ In the evolving landscape of stroke management, the advent of endovascular treatment (EVT) has marked a significant milestone, especially for acute ischemic stroke resulting from large vessel occlusions^2–5^. However, the application of EVT in patients with large ischemic cores remains a subject of debate and nuanced decision-making.

The study by Guo *et al*.^1^ assessed the efficacy of EVT in comparison to standard medical treatment (SMT) in patients with large ischemic cores, defined by an Alberta Stroke Program Early CT Score (ASPECTS) of ≤5. It was conducted across multiple centers in China and included a large cohort of 745 patients. The study’s results are both promising and cautionary. EVT, when combined with SMT, was associated with a higher rate of favorable functional outcomes compared to SMT alone^1^. Specifically, 36.9% of patients in the EVT + SMT group achieved favorable outcomes, compared to only 18.8% in the SMT group^1^. This significant difference underscores the potential of EVT to improve functional independence in this patient subset. However, this improvement does not come without increased risks. The incidence of symptomatic intracerebral hemorrhage (sICH) was notably higher in the EVT group (13.3%) compared to the SMT group (2.4%)^1^. This finding is a critical reminder of the inherent risks associated with EVT, particularly in patients with large infarcts, where the brain tissue is more vulnerable to injury and bleeding.

The challenge for neurosurgeons and stroke specialists lies in balancing these risks and benefits. While EVT can significantly enhance recovery prospects for some patients, it also exposes them to heightened risks of severe complications, as reported in a recent randomized trial by Costalat *et al*.^6^. The decision to opt for EVT requires careful consideration of various factors, including the patient’s overall health, the size and location of the infarct, and the time elapsed since stroke onset. The study by Guo *et al*. highlights that despite the increased risk of sICH, there was no significant difference in mortality between the EVT + SMT and SMT groups. This could indicate that while EVT increases hemorrhagic complications, it does not necessarily translate to higher mortality, possibly due to the improved functional outcomes in survivors. Moving forward, the field must focus on enhancing patient selection criteria for EVT in the context of large core infarcts. Advanced imaging techniques and biomarkers may play a crucial role in identifying patients who are likely to benefit from EVT without an undue increase in risk. Furthermore, developing protocols to minimize procedural risks and optimize post-EVT care will be essential.

In conclusion, while EVT opens a new frontier in the treatment of large core ischemic strokes, it requires a balanced approach to fully realize its potential. The journey of integrating EVT into broader clinical practice will undoubtedly be guided by ongoing research and a commitment to patient-centered care.

## Ethical approval

Not applicable.

## Consent

Informed consent is not applicable for this correspondence article.

## Sources of funding

Not applicable.

## Author contribution

O.O.O. and A.A.: conceptualization, project administration, supervision, validation, writing – original draft, and writing – review and editing; M.N.K., Q.S.Z., S.R., and P.S.: writing – original draft and writing – review and editing; A.O.A. and N.A.W.: supervision, validation, and writing – review and editing. All authors are accountable for all the aspects of this work.

## Conflicts of interest disclosure

No conflicts of interest to declare.

## Research registration unique identifying number (UIN)

Not applicable.

## Guarantor

Olabisi Oluwagbemiga Ogunleye.

## Provenance and peer review

Not commissioned, externally peer-reviewed.

## Data availability statement

Not applicable.
